# Hydrogels as Corneal Stroma Substitutes for In Vitro Evaluation of Drug Ocular Permeation

**DOI:** 10.3390/pharmaceutics14040850

**Published:** 2022-04-13

**Authors:** Susi Burgalassi, Erica Zucchetti, Leonardo Ling, Patrizia Chetoni, Silvia Tampucci, Daniela Monti

**Affiliations:** 1 Department of Pharmacy, University of Pisa, 56126 Pisa, Italy; erica.zucchetti@phd.unipi.it (E.Z.); l.ling@studenti.unipi.it (L.L.); patrizia.chetoni@unipi.it (P.C.); silvia.tampucci@unipi.it (S.T.); daniela.monti@unipi.it (D.M.); 2 Inter-University Center for the Promotion of the 3Rs Principles in Teaching & Research (Centro 3R), 56122 Pisa, Italy

**Keywords:** corneal stroma substituted, hydrogels, ocular permeation, beta-blockers

## Abstract

Hydrogels are complex hydrophilic structures, consisting of crosslinked homopolymers or copolymers insoluble in water. Due to their controllable bio-physicochemical properties mimicking the morphology of the native extracellular matrix, they are a key part of a lot of research fields, including medicine, pharmaceutics, and tissue engineering. This paper was focused on the preparation and characterization of hydrogels from different blends of polyvinyl alcohol (PVA) with microcrystalline cellulose (MCC) and gelatin (GEL) at various ratios, and from gelatin and chitosan alone to understand their feasibility of utilizing as corneal stroma substitutes in permeability tests for drug candidate molecules in early stages of their development. The characterization was carried out by differential scanning calorimetry, electron microscopy (SEM), water content, mass loss, water permeability, wettability, and tensile stress–strain tests. After the physicochemical characterization, PVA/MCC blend and chitosan proved to be the most promising constructs, showing negligible mass loss after immersion in aqueous medium for two weeks and low hydrodynamic permeability. They were then employed in drug molecules permeation studies and these data were compared to that obtained through excised tissues. The results obtained showed that PVA/MCC hydrogels have similar mechanical and permeability properties to corneal stroma.

## 1. Introduction

The development of new topical ophthalmic drugs and formulations necessitate performing permeation studies to evaluate the capability of compounds to pass through the cornea. Therefore, in vivo and ex vivo experiments are required. This leads to the employment of a large number of animals with high costs and ethical controversies. Thus, in recent years, many efforts to develop an alternative model to reduce the animal tests were performed by researchers.

The cornea is a strong, highly transparent, flexible, and avascular connective tissue that occupies approximately 15% of the surface of the fibrous layer of the eye. It is a rigid organized structure containing water, proteoglycans, cells, and densely woven collagen fibrils as major structural components, and is formed by five distinct layers: epithelium, Bowman’s layer, stroma, Descemet’s layer, and endothelium [1,2].

The main barrier to transcorneal penetration is the epithelium, but the corneal stroma also plays an important role. In fact, the epithelial model in permeation studies has a good correlation with in vivo experiments only for hydrophilic and moderately lipophilic compounds. Instead, highly lipophilic substances show a higher permeation coefficient with respect to the entire cornea due to the absence of hydrophilic stroma. Therefore, the presence of stroma in the permeation model is essential in order to obtain permeation data for both hydrophilic and lipophilic compounds similar to original tissue [3].

The stroma, constituting about 90% of the corneal thickness, contains a high percentage of water, 65% of which is in the extracellular matrix (ECM) and 11% as cellular content. This water content plays an important role as an appropriate level of hydration is required to keep the tissue transparency. In addition to water, the stroma consists of collagen fibrils lamellae oriented parallel to each other and to the corneal surface [4]; the basic cementing substance is constituted by proteoglycans and between the lamellae, the keratocytes are located—these are stromal fibroblasts which are responsible for the production and maintenance of the ECM [5,6].

The fact that the corneal stroma is a network of fibers bearing a large amount of water is one of the justified reasons why hydrogels have become attractive structures for the development of the entire cornea construct.

Numerous types of hydrogels can be produced as a result of a wide range of suitable starting materials and a broad variety of chemical or physical methods. Both natural and synthetic polymers have been used to obtain hydrogels with different biomedical applications.

There is a wealth of literature present on hydrogels that are potentially suitable for many ophthalmic applications excluding drug delivery systems, and the topic is still currently under-researched. In 1999, Griffith constructed a functional human corneal equivalent based on a collagen-chondroitin sulphate substrate [7]. Subsequently, the collagen (crosslinked using 1-ethyl-3-(3-dimethylaminopropyl)carbodiimide (EDC) and N-hydroxysuccinimide (NHS)) and various synthetic polymers were blended to fabricate corneal substitutes [8]. In line with the examples aforementioned, other investigations reported on collagen-based film reinforced by surface grafting of chondroitin sulphate showing an excellent biocompatibility, with a good proliferation of human corneal epithelial cells [9] or collagen-chitosan mixtures crosslinked by alginate dialdehyde as membranes with an improved mechanical properties and good biocompatibility [10].

Other materials based on gelatin, keratin, chitosan, and silk have also been used for corneal tissue engineering. Porous membranes of gelatin coupled chemically with chondroitin sulphate by using EDC-NHS exhibited great potential as they could achieve enhanced permeability and keratocyte adhesion with a stimulatory effect on the proliferation and biosynthetic activity of corneal stromal cells [11]. Gelatin–collagen composites as hydrogels crosslinked by EDC-NHS also might represent potential candidates for future corneal applications considering their adequate mechanical properties, optical transparency, and cytocompatibility [12].

Chitosan is another polymer that has raised a lot of interest. Widely used as wound dressing biomaterials in clinical practice, it has also been shown that synthetic chitosan membranes can support the growth of cultured corneal epithelial cells in good condition with minimal toxicity [13]. Chitosan has also been blended with collagen and hyaluronic acid to mimic the collagen–glycosaminoglycans scaffolding of the extracellular matrix in various tissue engineering applications, including scaffolds for corneal applications [14]. The same pair, EDC-NHS, as the coupling agent was used to fabricate chitosan-based scaffolds with controllable mechanical properties, optimal strength, elasticity, and optical transparency for corneal tissue engineering [15,16]

Cellulose is usually employed together with other polymers to form hydrogels with enhanced properties: polyvinyl alcohol (PVA) is one common polymer that is paired with it due to its high mechanical strength and elasticity. PVA/cellulose hydrogels are synthetized via both chemical and physical methods [17]: the former involve the use of crosslinking agents such as epichlorohydrin, the latter use freezing and thawing cycles. The physically crosslinked hydrogels have strong interactions between the two polymers, leading to a dense structure and high strength. PVA/cellulose hydrogels do not affect the growth of corneal epithelial cell cultures, supporting the non-cytotoxic characteristics of the blend, and viable cells can adhere to it [18].

However, the use of these hydrogels for permeation studies of ophthalmic drugs has not been investigated.

This work aims to select materials and methods that could be used to develop and construct a corneal stroma-like hydrogel that might be used as a substitute for animal corneas in drug testing, specifically for ophthalmic drug permeation.

A stroma equivalent that imitates ocular tissue permeability with regard to membrane microstructure and physicochemical properties could also provide valuable information for the development of innovative means to improve ophthalmic drug delivery.

## 2. Materials and Methods

### 2.1. Materials

Polyvinyl alcohol (PVA; PVA 26-88, Merck, Darmstadt, Germany), cellulose microcrystalline (MCC; Pharmacel 102-M, DFE Pharma, Nörten-Hardenberg, Germany), gelatin (GEL; 120 Bloom, porcine gelatin Type A, A.C.E.F. SpA, Fiorenzuola D’Arda, Italy), chitosan (CHIT; powdered shrimp, 87.6% degree of deacetylation, Marine Chemicals, Mumbai, India), timolol maleate (TM), penbutolol sulphate (PB), atenolol (AT), and betaxolol hydrochloride (BX) (Sigma-Aldrich, Milan, Italy).

All other chemicals and solvents were at least of an analytical grade.

Ringer–Krebs buffer solution (RKB) had the following composition: 5.6 g/L sodium chloride, 0.34 g/L potassium chloride, 0.047 g/L magnesium chloride hexahydrate, 1.8 g/L D-glucose, 2.0 g/L sodium phosphate monobasic monohydrate, and 3.6 g/L sodium phosphate dibasic anhydrous.

### 2.2. Preparation of Hydrogels—A Brief Approach

Numerous types of hydrogels applying different crosslinking techniques to different starting materials (PVA, cellulose, gelatin, and chitosan) were produced since we hypothesized that these materials, as well as their combination, could lead to composites with desirable mechanical properties.

Polyvinyl alcohol is a water-dispersible and biocompatible synthetic polymer, able to form films resistant to solvents, with a high flexibility and tensile strength and a low tendency for protein adhesion. These properties are dependent on the degree of moisture, since water can act as a plasticizer, reducing the tensile strength and at the same time increasing the tear strength, thus making it suitable for a broad variety of pharmaceutical applications.

PVA, by itself or blended with other substances, such as cellulose derivates, chitosan, or gelatin, has been used to produce hydrogels and films through both chemical and physical crosslinking methods [1,17]. We chose to use blends of PVA with cellulose (MCC) or gelatin to obtain hydrogels by freezing/thawing cycles: cellulose was chosen due to its biocompatibility, good mechanical properties, and its ability to modify and enhance its porous structure through reactions and crosslinking with other polymers [17,19,20]; gelatin was chosen because of its ease of processing and large availability.

Due to the low water solubility, MCC was dissolved using an aqueous solution of sodium hydroxide/urea followed by cooling, according to Cai et al. [19].

Gelatin-based materials have shown good transparency due to their high-water content and biocompatibility, so they are widely used for ocular applications, in tissue engineering, and cell culture. The chemical or physical crosslinking of gelatin, alone or in combination with other polymers, has produced a wide range of scaffolds with different physico-chemical properties suitable in ophthalmic surgical procedures or as candidates for corneal tissue engineering. For example, both chemical and physical crosslinking can be achieved through EDC-NHS coupling reactions and via ultraviolet light irradiation and dehydration [1,11,21,22].

Chitosan is the second most abundant polymer in nature after cellulose; it is biocompatible, biodegradable, similar to most of the materials employed to produce hydrogels for pharmaceutical and biomedical use, and has antimicrobial and wound-healing properties [13,23]. Its crosslinking can be produced by pH changes or phase separation in a nonsolvent, methods that do not residue toxic agents, but also using chemical agents, as glutaraldehyde or benzaldehyde, and physical treatments; for example, the UV crosslinking approach can be used to produce chitosan scaffolds with mechanical properties applicable as biomedical devices [16,24,25,26].

The hydrogel compositions, as well as their obtaining methods, are listed in Table 1.

#### 2.2.1. Freezing/Thawing Method

The hydrogels were obtained using PVA in combination with MCC or GEL, physically cross-linked by the freeze–thaw technique [17]. The solutions were prepared separately: (i) MCC was dissolved into an aqueous solution of urea (4% *w/w*) and sodium hydroxide (6% *w/w*) with stirring for about 10 min to form a 4% *w/w* dispersion. It was then stored in a refrigerator overnight. The frozen material was then warmed up at room temperature (20–22 °C) and stirred until the solution appeared transparent. (ii) PVA was dissolved in water under stirring at 90 °C; it was then blended with a mixture of urea and sodium hydroxide in an aqueous solution to obtain a final PVA concentration of 4% *w/w* in a solution with the same concentration of sodium hydroxide and urea of solution described in (i). (iii) GEL was dissolved in water under stirring at 40 °C to obtain a 10% *w/w* dispersion.

The dispersions were then mixed in different PVA/GEL and PVA/MCC ratios: 90:10, 80:20 and 70:30 (*w/w*).

**Figure 1:**
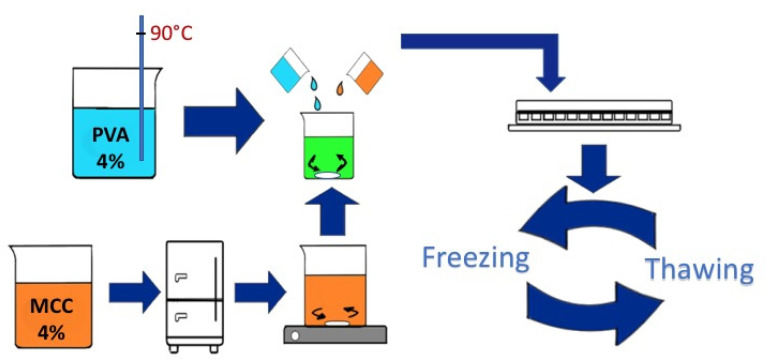
Preparation scheme of PVA/MCC hydrogels.

PVA/MCC and PVA/GEL dispersions were placed into 24 wells-plate and subjected to 7 or 14 freezing/thawing cycles (Table 1). In Figure 1, a scheme of the preparation of PVA/MCC hydrogels is shown.

The hydrogels were compressed between two glass panes under a weight until a thickness of 0.6 mm was achieved and then washed with deionized water until neutral pH to remove the excess of the NaOH/urea mixture.

#### 2.2.2. Chitosan Hydrogel Obtaining

Chitosan solutions at a concentration of 4% were prepared by dissolving the appropriate amount of deacetylated chitosan in a 2% acetic acid solution at 60 °C under stirring. The resulting product was filtered using a sieve mesh and poured into 24 wells-plates, and sodium hydroxide 0.5 M was subsequently added. After 30 min of contact, the hydrogels were washed with deionized water up to about pH 7 (Table 1) [13].

#### 2.2.3. UV Irradiation

A solution was prepared by dissolving at 70 °C by stirring GEL and glucose in water in a 2:1:2 ratio. The product was then poured into a glass container and exposed to UV irradiation (254 nm, 33 W) overnight (Table 1).

### 2.3. Characterization of Hydrogels

#### 2.3.1. Mass Loss, Erosion, or Dissolution

In order to verify the possibility to employ the hydrogel as a substrate for ophthalmic drug permeation evaluation, the resistance in aqueous medium was verified.

The samples of dried hydrogel were weighted (*W_d0_*) then submerged in about 15 mL of RKB and maintained in incubation at 37 °C for 15 days. After this period, they were dried at room temperature (20–22 °C) to constant weight (*W_d1_* = final dry mass) and the *mass loss* was determined by the following equation:

*Mass loss*% = 100 × (*W*_*d*0_ − *W*_*d*1_)/*W*_*d*0_ (1)

The experiment was carried out in triplicate.

#### 2.3.2. Water Content

The hydrogels were maintained in water for 24 h at room temperature (20–22 °C), then their surface was wiped with filter paper to remove the excess of water, and finally they were weighted. After that, the samples were dehydrated at room temperature (20–22 °C) until constant weight and weighted again. The water percentage (*water*%) and the specific water content (*S*) of hydrogels were calculated as follows:

*Water*% = 100 × (*W*_*h*_ − *W*_*d*_)/*W*_*h*_ (2)

*S* = (*W*_*h*_ − *W*_*d*_)/*Aσ* (3)

where *W_h_* is the weight of the hydrated hydrogel, *W_d_* the weight of the dried hydrogel, *A* and *σ* are the base area and height of the cylindrical hydrated hydrogel, respectively. The diameter and height of the hydrated hydrogels were measured using a digital microscope (Dino-Lite 2.0., Dino-Lite, Taiwan).

The experiment was carried out in triplicate.

#### 2.3.3. Contact Angle Measurements

The wettability characteristics of the hydrogel by water were determined by contact angle goniometry using the captive bubble technique, where the measurement of the contact angle values of an air bubble placed on the surface of a material that has reached a swelling equilibrium was carried out [27,28].

The measurements in this study were performed at 24 °C with an OCA15 optical contact angle measuring system (DataPhysics Instrument, Filderstadt, Germany). The system consisted of a high-resolution CCD video camera and a six-fold power zoom lens with integrated fine focusing; the images were recorded and analyzed by SCA20 software (DataPhysics Instrument, Filderstadt, Germany).

**Figure 2:**
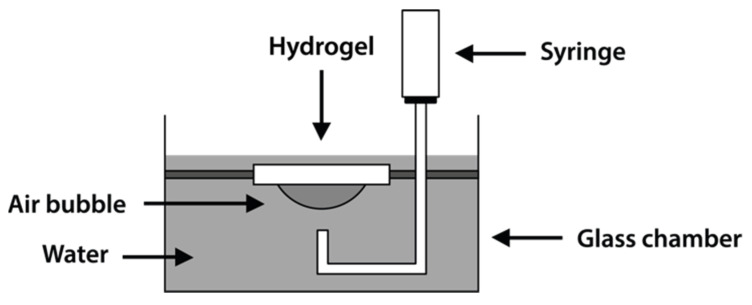
Schematic diagram of the apparatus for contact angle measurements of air on hydrogels by the captive bubble technique.

The gel was carefully suspended in a clear glass chamber full of Milli-Q water by a holder built for this purpose; a small air bubble of 3 µL was dispensed and placed on the bottom side of the gel by the means of a curved capillary tube applied to a syringe (Figure 2). The image of the bubble was then captured, and the mean contact angles were derived from each picture. In order to obtain a reliable value and to reduce the variability caused by the employed technique, each sample was measured 12 times; for every type of hydrogel, 10 different samples were employed.

#### 2.3.4. Differential Scanning Calorimetry (DSC)

DSC measurements were performed by Pyris DSC 6 (Perkin Elmer, Milan, Italy) and its relative software (Pyris Instrument Managing Software, Version 3.8, Perkin Elmer, Milan, Italy) was used for the acquisition and processing of the data. The temperature and energy scales of the instrument were calibrated using indium and zinc references.

About 3 mg of dried material were weighed and incapsulated in pans which were then placed in the sample holder alongside the empty reference pan. The carrier gas was nitrogen, with a flow rate of 20 mL/min. The runs were performed between 30 °C and 300 °C, with a heating rate of 10 °C/min. The melting temperature was determined by the peak position, while the enthalpy (heat) of fusion (ΔH) was determined by integrating the area under the corresponding endothermic peak.

Each analysis was performed in triplicate.

#### 2.3.5. Tensile Strength

Tensile tests are commonly used to evaluate mechanical properties of hydrogels, such as toughness and mechanical strength. These characteristics are becoming more relevant as the interest in producing hydrogels with a significant mechanical performance increases.

**Figure 3:**
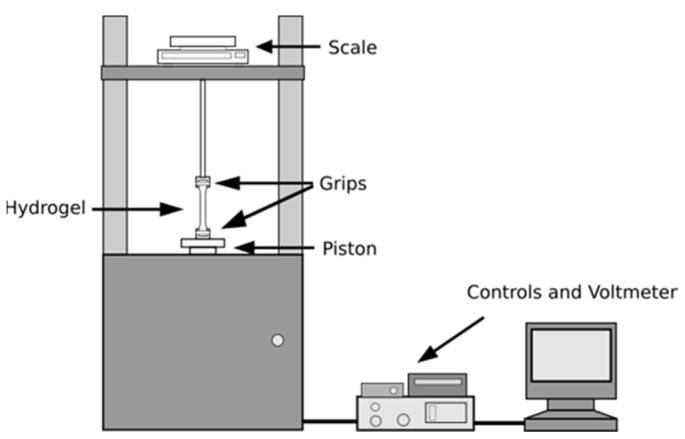
Schematic diagram of the apparatus for measurements of tensile strength.

Large-strain tests were carried out using a mechanical testing machine (Figure 3), consisting of:

- A balance at the top, connected to the upper grip, which outputs an electric signal related to the force applied to the sample by the machine;
- A piston, connected to the lower grip, which outputs an electric signal related the sample elongation caused by the stress;
- A voltmeter (Handyscope HS2, TiePie Engineering, Sneek, Holland) which, with its channels linked to both piston and scale, records the input signals coming from them, digitizes (carried out with an analog-to-digital converter, in a process called quantifying) and processes the values, and displays them as a digital data.

The accompanying software of Handyscope HS2 (TiePie Engineering, Sneek, Holland) was used to obtain the strain and stress values over time.

Dog bone-shaped gel strips were cut from sheets of hydrated hydrogel using a stencil; the samples had a total length of 42 mm with a gauge length of 14 mm, width of 4 mm and their thickness (about 0.6 mm) was exactly measured using a digital microscope (Dino-Lite 2.0., Dino-Lite, Hsinchu, Taiwan) in order to calculate the real value of the cross-section area. The strips were placed in position between two grips and a tensile force with a constant ramp rate of 3.75 mm/min was applied until the hydrogel fractured. The data were processed only if the fracture occurred in the narrow part of the strip.

The values of the apparent Young’s modulus (*E*) were calculated by:

*E* = *σ*(*ε*)/*ε* = (*F*/*A*)/(∆*L*/*L*_0_) = (*FL*_0_)/*A*∆*L* (4)

where *σ*(*ϵ*) is the tensile strength, *ϵ* is the extensional strain, *F* is the force applied to the sample, *A* is the cross-section area perpendicular to the applied force, ∆*L* is the change in sample length, and *L*_0_ is the initial gauge length of the sample.

Each experiment was performed in ten-fold.

#### 2.3.6. Scanning Electron Microscopy Analysis

SEM analysis was used to examine the morphology of PVA/MCC hydrogels after freeze-drying by employing a scanning electron microscope (Phenom XL G2 Desktop, Alfatest, Rome, Italy). After an hour in deionized water, the samples were maintained at −80 °C all night, then they were lyophilized. The dry samples were examined with SEM under high vacuum conditions by using an accelerating voltage of 5 kV.

#### 2.3.7. Hydrodynamic Permeability

**Figure 4:**
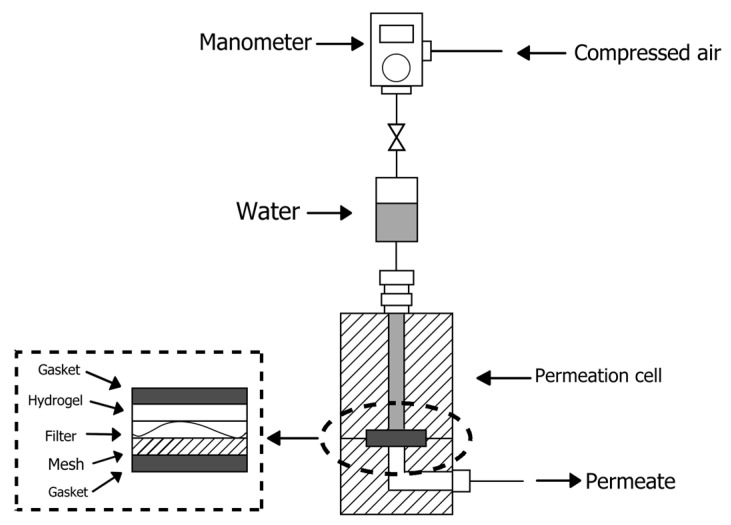
Schematic diagram of apparatus for the determination of hydrodynamic permeability.

The Darcy permeability of the hydrogels to water was evaluated using a custom-made apparatus, schematized in Figure 4. The sample was mounted in a holder sandwiched between two hemi-cells. The holder, starting from the origin of the water then moving towards the exit side, consisted of:

- A gasket with an opening in the center to help keep the hydrogel in the correct position;
- A filter paper to separate the hydrogel from the contact with the metal mesh. Its resistance to the passage of water was negligible;
- A metal mesh to avoid the probable deformation of the hydrogel due to the applied pressure;
- Another gasket, identical to the other, to control the surface area of the hydrogel participating in the permeation.

The hydrogel sample was placed between the first gasket and the filter paper, as shown in the picture.

Each hemi-cell has an aperture: the upper one used for the inlet of pressurized water and the lower one used to collect the water permeated through the hydrogel by a capillary tube. The cell is linked to a pipe filled with water that is connected to a compressed air system. The output of the line is equipped with a manometer that allows the regulation of the pressure to be applied to the water and, therefore, to the hydrogel.

The experiment was repeated at least five times for each type of hydrogel, and the hydraulic permeability (*K_m_*) was calculated using the equation:

*K*_*m*_ = (*η*
*V*_*m*_
*L*)/∆*P* (5)

where *η* is the viscosity of water, *V_m_* is the water flow rate per the unit of hydrogel area, *L* is the thickness of the hydrogel, and ∆*P* is the pressure difference of the water, as reported by Kapur and colleagues [29].

The thickness of the hydrogel (*L*) was determined by interposing it between two glass panes of a known thickness and measuring the total thickness by a micrometer.

### 2.4. Permeation Studies of Molecules across Hydrogels

The permeation studies through the hydrogels were performed with a series of beta-blocking agents with a homologous structure and different degree of hydrophilicity—atenolol, betaxolol hydrochloride, penbutolol sulphate, and timolol maleate—using horizontal permeation cells. The cells were composed of two parts, a donor and a receiving compartment. The hydrogels were mounted between the compartments and one side was filled with the drug solution (the donor); meanwhile, the other side contained the Ringer–Krebs buffer (RKB, the receiving phase). The process was carried out at a constant temperature of 32 °C, using a temperature-controlled water bath.

At appropriate time intervals, 1 mL of the receiving solution was withdrawn and replaced with the same volume of fresh RKB. Each experiment was conducted for 4 h and was performed in triplicate.

The withdrawn volume was analyzed by HPLC using a LC-20AT system equipped with an SPD-10A UV detector, a CBM-20A interface (Shimadzu, Kyoto, Japan), and a 20 μL Rheodyne injection valve. The analyses were conducted with a C18 Bondclone (10 μm, 300 × 3.9 mm; Phenomenex, Torrance, CA, USA) column and the conditions are listed in Table 2.

From the obtained data, the apparent permeability coefficient (P_app_) values were calculated according to Fick’s first law.

### 2.5. Statistical Analysis

Statistical significance between 2 groups was analyzed by Student’s *t*-test while a Kruskal–Wallis nonparametric test, followed by Dunn’s test, was used for multiple comparisons. At least a *p*-value < 0.05 was considered statistically significant. All data processing was performed using Prism software, version 8.0 (GraphPad Software Inc., San Diego, CA, USA).

## 3. Results and Discussion

### 3.1. Characterization of Hydrogels

#### 3.1.1. Mass Loss and Water Content

A feature necessary for our purpose was the ability of the hydrogel to withstand an aqueous medium for the period of time required for the stratification of the epithelial cells.

Thus, as a first step, the capability of the structures to resist for two weeks whilst immersed in water at 37 °C without undergoing significant erosion or dissolution was investigated. Unfortunately, the gelatin that resulted was unsuitable to our purpose as, after a few minutes in water at 37 °C, most of the samples dissolved. This behavior can be attributed to an extensive amount of gelatin that was not successfully crosslinked after the UV exposure, or dehydration that led to the materials lacking the desired characteristics [22].

The PVA/gelatin hydrogels were also unsuitable: in fact, although the system was still present after the period of incubation in aqueous medium, the calculation of mass loss showed that a quantity of material similar to the gelatin percentage was lost (Table 3). These results suggest that the gelatin was not crosslinked and, therefore, could be entirely dissolved.

For this reason, the gelatin structures were not subjected to subsequent studies.

Instead, the PVA/MCC and chitosan hydrogels showed quite low mass loss, attaining a maximum value of 3.7% for PVA/MCC-7 and 2.2% for PVA/MCC-14 (Table 3). As described by Hassan and Peppas [30], it is apparent that repeated freezing/thawing cycles reinforce the hydrogel structure via PVA crystals (generated during freezing) by which these systems are physically crosslinked. This could explain the tendency of the 7 cycle-treated hydrogels to lose a greater percentage of mass over long periods of time compared to the 14 cycle-treated ones.

Moreover, PVA/MCC hydrogels showed water content of about 82–85% (Table 3), very close to the normal hydration levels of the native corneal stroma, which is about 76–80% [31,32]. Instead, chitosan hydrogel showed a greater percentage of water that was about 90%, significantly different from all other hydrogels tested.

#### 3.1.2. Contact Angle Measurements

This procedure was deemed preferable since the captive bubble method showed high repeatability [33] without being too affected by environmental factors, such as temperature or pressure. However, given the statistically different results obtained by changing software or procedure, those authors suggest that different techniques and methods could give variable results. On the other hand, the main limitation of the sessile drop method is the dehydration of the hydrogel, coupled by variations induced from environmental factors. Additionally, since wetting is not a static condition, the air bubble can be in numerous stable metastates, causing the high variability in the measured values. Furthermore, as the method utilizes image processing to measure contact angles, it is inherently prone to errors due to machine and sensor limitations, as well as the image resolution being able to cause errors that can reach and sometimes exceed 10° [34].

The results of the contact angle measurements are listed in Table 4.

PVA/MCC hydrogels showed a reduction in the contact angle values with increasing cellulose concentration in both 7 and 14 cycle samples, from 42.42 ± 0.70 to 33.13 ± 0.60° and from 41.72 ± 0.64 to 34.50 ± 0.40°, respectively, meaning an increase in hydrophilicity corresponding to an increase in the amount of cellulose in the hydrogel. The increase in cellulose content can act on two fronts, which both lead to an increase in hydrophilicity: its presence in higher percentage and an increase in PVA crystallinity, after several freezing and thawing cycles, as reported by Shalom et al. [35]. This increase in crystallinity corresponds to an increase in hydrophilicity and a decrease in contact angle, a trend that can be seen going from the 90:10 to the 70:30 PVA/MCC hydrogels [36]. The contact angle values obtained from the three ratios of PVA/MCC were statistically different, instead no significant differences between the values measured for hydrogels with the same percentage of compounds and different number of freeze/thaw cycles were shown. Therefore, the wettability values of the samples did not seem to be influenced from the number of cycles; hence, by the structure assumed by the hydrogel—but only by the composition—the PVA:MCC ratio was obtained. The contact angle value of the chitosan scaffold was about 35°, an average value among those measured on the PVA/MCC hydrogels. Compared to other published data, the hydrogel wettability both for PVA/MCC and chitosan was slightly higher than for the stroma (contact angle about 44°) [37] or the cornea (50 ± 5°) [38].

#### 3.1.3. DSC

**Figure 5:**
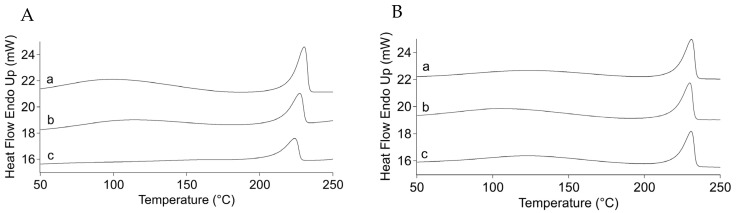
Thermograms of PVA/MCC-7 (**A**) and PVA/MCC-14 (**B**) hydrogels with (a) 90:10, (b) 80:20, and (c) 70:30 ratios.

In order to evaluate the differences between the various PVA/MCC mixtures from a thermal behavior standpoint, the PVA/MCC hydrogels were subjected to calorimetry studies. The thermograms of PVA/MCC-7 and -14, reported in Figure 5, have shown a tendency to increase in enthalpy (heat) of fusion (ΔH) values with an increasing PVA content, from 63.43 ± 0.50 to 81.14 ± 0.60 J/g and from 63.97 ± 4.38 to 70.27 ± 0.28 J/g, respectively (Table 5). Similar results were reported by Chang and colleagues [17] who observed an increment in both the melting temperature and ΔH values for their samples of PVA/cellulose 25:75, 50:50, and 75:25 ratios that caused a shift from 231.9 °C to 237.7 °C in melting temperature and from 17.57 to 55.92 J/g in ΔH values. The authors suggested that these variations were derived from the high crystallinity and thermal stability of PVA. In our case, the change in melting temperature was not noticeable, probably due to a smaller difference in PVA percentage between samples compared to the ones employed in the study of the literature, but an ascending tendency in the values of the heat of fusion with increasing PVA content was evident.

#### 3.1.4. Tensile Strength and Young’s Modulus

**Figure 6:**
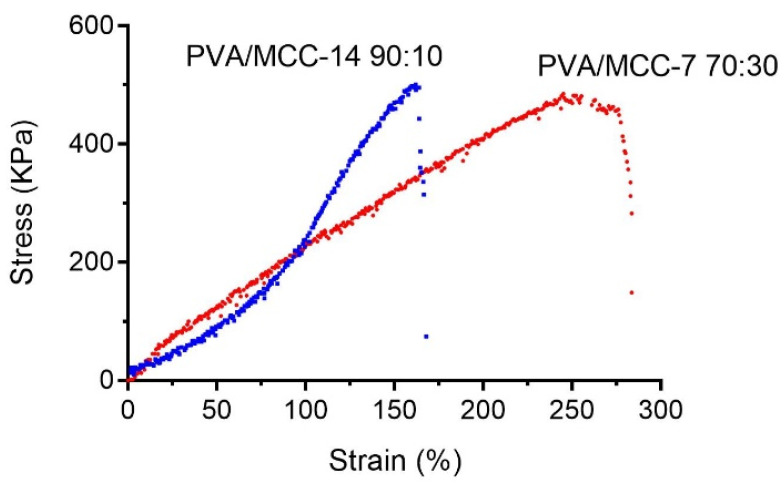
Typical tensile stress–strain curves of PVA/MCC hydrogels.

The results of the analysis of the mechanical parameters of hydrogels are listed in Table 6, while an example of the typical stress–strain curves recorded during the measurements are shown in Figure 6.

A tendency can be observed as the increase in cellulose correlates to an increase in both tensile strength and Young’s modulus in the 7 cycle-treated hydrogels; while in the 14 cycle-treated ones, it correlates to a decrease in these values. Such behavior might be attributable to a reinforcement effect that the cellulose has towards the PVA hydrogels [39,40,41] and to the increase in the number of freeze–thaw cycles [42,43,44]. In fact, it is reported that PVA-based hydrogels, irrespective of the preparation methods, become more resistant when cellulose is added, even in low amounts. Our results show a gradual increase in tensile strength from 249.10 ± 8.56 to 638.21 ± 34.12 kPa as the cellulose content in PVA/MCC-7 hydrogel increased from 10% to 30%. With the increment of the cellulose incorporated, the elastic modulus also increased by a factor of 2.17, from 84.21 ± 3.23 to 182.70 ± 9.18 kPa. Ren and collaborators attributed this behavior to the high strength of the compact structure of the cellulose network and to the beneficial interaction at the interface between the cellulose and PVA network [40]. This results in the loss of intramolecular interactions of PVA in favor of intermolecular cellulose-PVA interactions. The formation of these new interactions then strengthens the hydrogels. It is also true, however, that the strength of PVA-based hydrogels does not increase indefinitely as the number of freeze–thaw cycles increases—after six, it reaches a plateau when assessed with both tensile and compressive tests [44,45]. In fact, each cycle causes a remodeling in the PVA structure due to ice formation that produces regions with higher concentration of polymer. It could then be hypothesized that the high number of freeze–thaw cycles change the microstructural rearrangement of the PVA due to hydrogel phase separation that cause an increased density of regions rich in water and polymer. Furthermore, this process is believed to push the PVA chains closer to each other, facilitating the formation of crystallites and permanent physical cross-linkages inside the hydrogel network. In this hypothesis, cellulose produces fewer interactions with PVA despite its increased content. Therefore, the strength of the hydrogel remains, de facto, anchored only to the cross-linking of the PVA which, decreasing in concentration, produces less strong hydrogels.

The chitosan scaffold showed values of 110.51 ± 19.33 kPa for elastic modulus and 163.89 ± 32.2 kPa for tensile strength, the lowest of the values measured.

Data on the tensile strength of the corneal stroma are reported in the literature, but these often have a high variability. Zhang et al. [46] obtained 1530 ± 860 and 550 ± 220 kPa of tensile strength values and Young’s modulus values of 1160 ± 1420 kPa and 1410 ± 1350 kPa for anterior and posterior stroma, respectively. Other authors observed much lower elastic modulus values for anterior and posterior stroma, ranging from 245.9 ± 209.1 to 281 ± 214 kPa and from 100.2 ± 61.9 to 89.5 ± 46.1 kPa, respectively [47,48].

#### 3.1.5. Permeability Coefficient and Microstructural Arrangement

The most important property for the proposal of this research was the permeability coefficient. In fact, the goal was to produce a structure able to substitute the excised cornea in the preliminary permeation studies of drugs from formulations to reduce the use of animals in experimentation.

PVA/MCC structures showed very different hydraulic permeability when subjected to 7 or 14 freezing/thawing cycles, from 2.72 × 10^−14^ to 6.61 × 10^−14^ cm^2^ and from 31.00 × 10^−14^ to 64.61 × 10^−14^ cm^2^, respectively (Table 7).

Since the permeability is a property which depends on the amount of void spaces (pores) present in the hydrogel which the fluid can pass through, the highest values measured for the 14 cycle-treated hydrogels compared to the 7 cycle-treated ones can be explained by the increase in hydrogel pore size following an increase in the number of freeze–thaw cycles. This correlation was also observed by Holloway and colleagues [45] who attributed this change to the microstructural rearrangement of the PVA, as previously discussed in Section 3.1.4.

**Figure 7:**
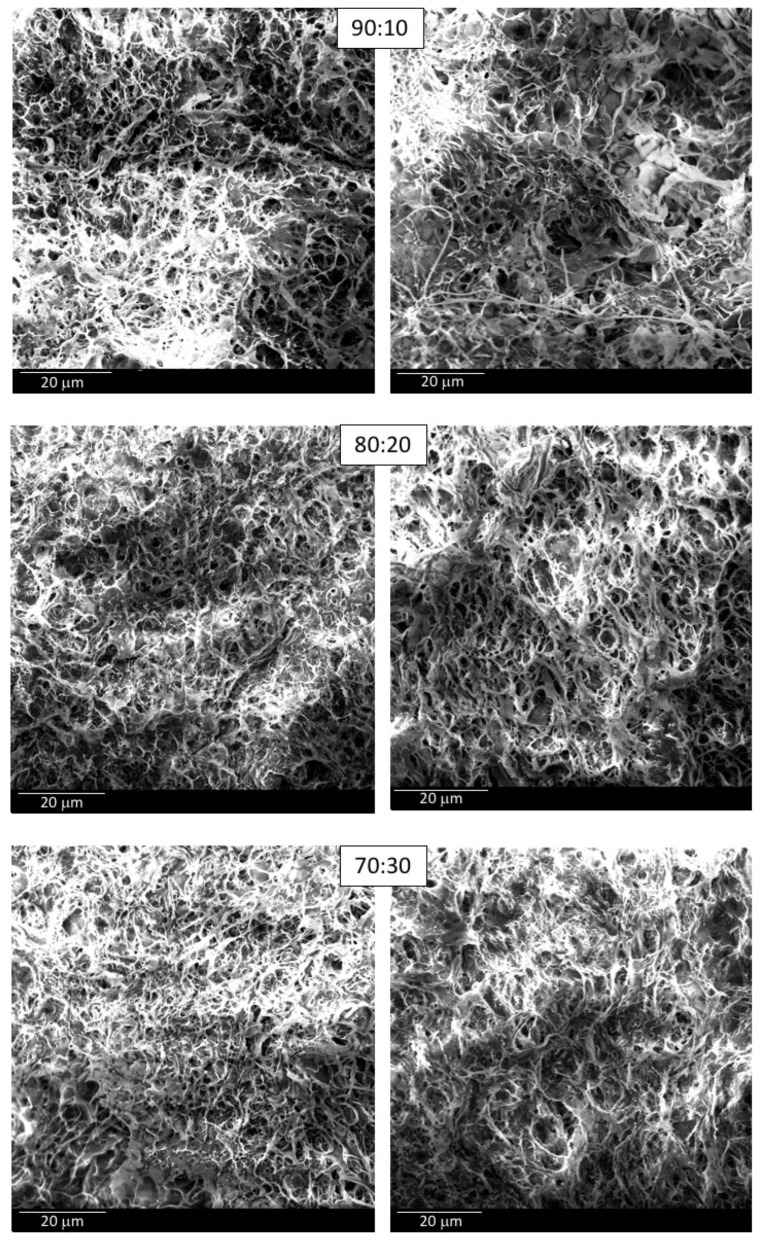
SEM imagines of PVA/MCC-7 (on the left) and PVA/MCC-14 (on the right) hydrogels at different PVA/MCC ratios.

The images obtained by SEM (Figure 7) seem to support this thesis, showing a certain variability in the pore size of the different hydrogels, albeit not so markedly.

The values of the specific water content calculated for all the PVA/MCC hydrogels (Table 7) do not show such differences that could suggest their different porosity. However, it is important to remember that not all of the space defined by the porosity of a saturated porous+ structure is available for permeation, considering that the content of water adsorbed onto the pore surfaces and that pores are often tortuous, intertwined, and dead-end. Determining exactly the average pore diameter of a hydrogel is not simple as there are many factors to take into account that can come into play during the measurement [49]. Many theoretical approaches have been proposed which lead back to pore size by evaluating the permeability and the porosity of a porous structure, such as hydrogels. In any case, the proposed equations contain a constant typical of the geometry of the system, including the tortuosity of the pores, which is not always known and requires skill in its determination. On the basis of a model developed by Ferry [50], Refojo [51] determined this constant to be equal to eight for certain hydrogels by giving the equation:

*r* = (8 *k_m_*/*S*)^1/2^ (6)

where *r* is pore radius and *k_m_* and *S* were already defined earlier.

For comparative purposes only, this equation was used to calculate the pore size of the investigated hydrogels (Table 7) although we are aware that the constant may not fit the characteristics of our constructs but correlates with the two parameters used for the calculation.

**Figure 8:**
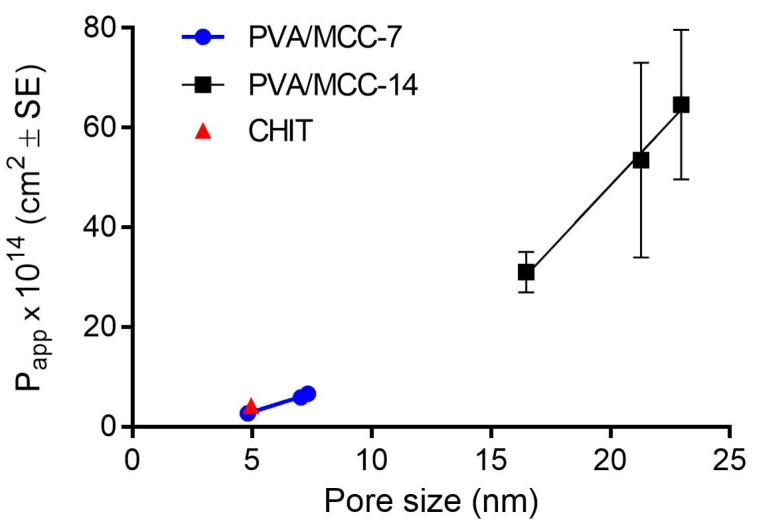
Correlation of the permeability coefficient as a function of the pore size for the hydrogels under study. For some points, the error bar is shorter than the height of the symbol.

As well as being predictable from theoretical calculations, the larger the pore radius, the larger the apparent permeability. The good correlation of the data is also shown graphically in Figure 8, where P_app_ values are reported as a function of the calculated pore size.

PVA/MCC-7 and chitosan scaffolds showed very similar permeability coefficient values but significantly different specific water content, tracing what has already been seen in the water content percentage determination.

Moreover, it should be pointed out that PVA/MCC-7 and CHIT hydrogels had permeability coefficient values of the same order of magnitude as those of corneal stroma at normal hydration levels, about 1.36 × 10^−14^ cm^2^ when normalized for water viscosity at room temperature [51].

Regarding the microstructure of PVA-based hydrogels, it can also be seen from Figure 7 that significant differences related to the different ratios of PVA and MCC are not appreciable; in fact, all the hydrogels showed high porosity with no preferential orientation of the solid structure.

### 3.2. Permeation Studies

Based on the results obtained and discussed so far, PVA/MCC-7 and CHIT hydrogels appear to be the best candidates to simulate the corneal stroma and, therefore, have been used for an initial test of permeability evaluation of a homologous series of beta-blocking drugs with comparable molecular weights, chosen to have different degrees of hydrophilicity (Table 8).

**Figure 9:**
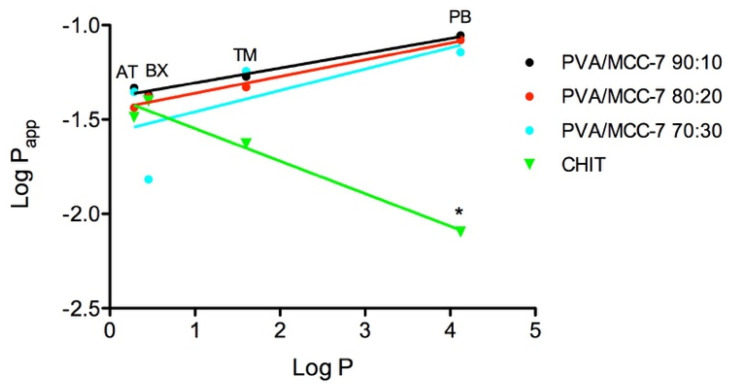
Relationship between the logarithmic values of partition coefficient (P) of the model drugs and their apparent permeability (P_app_) across different hydrogels. AT: atenolol; BX: betaxolol; TM: timolol; PB: penbutolol. * Statistically different from the other P_app_ values of BX.

Figure 9 illustrates a graph of P_app_ vs. P, both expressed as logarithmic values, for the beta-blockers under study. The figure shows a good linear relationship (R^2^ = 0.97 and 0.98; Table 9) of the parameters for all hydrogels employed, except for PVA/MCC-7 70:30. It can be seen that there is a trend towards a decrease in the permeability of the molecules as the amount of PVA present in the PVA/MCC-based hydrogels decreases. It is also evident that the Log P_app_ values tend to decrease with the increase in the Log P value when CHIT hydrogel is used as a barrier. However, it should be specified that the one-way ANOVA test did not show any differences between the data series obtained for each hydrogel. A statistically significant difference between CHIT and the PVA-based hydrogels was found only on the P_app_ values obtained with penbutolol when an unpaired *t*-test was applied (maximum *p* = 0.003 versus PVA/MCC 90:10).

The different behavior highlighted towards CHIT could be due to the higher water content of this hydrogel compared to those based on PVA; in fact, the content in CHIT hydrogels was found significantly higher than that of PVA/MCC-7 and penbutolol is the molecule with the highest partition coefficient. From the literature data, it is known that the P_app_ through the corneal stroma is minimally influenced by the partition coefficient of the solute, even when this varies by several logarithmic units [32,53,56], and this scarce influence is also demonstrated by low slope values of the LogP_app_/Log P correlation lines (Table 9). However, regarding CHIT hydrogel, it can be hypothesized that a water content higher than that physiologically present in the corneal stroma may actually slow down the permeation of highly lipophilic molecules.

Regarding the P_app_ values resulting from permeation experiments of beta-blockers across corneal stroma, Huang and colleagues [32] found that 12 drugs with a four-fold logarithmic interval of partition coefficient displayed almost constant permeability coefficients across the stroma, about 12 × 10^−2^ cm/h. Sasaki’s research team reported the same trend, but with lower P_app_ values (from 5 to 9 × 10^−2^ cm/h), much closer to what we obtained in our study across the hydrogels [53].

## 4. Conclusions

In this study, we developed several types of hydrogels based on cyto-compatible and inexpensive polymers which can be produced by simple procedures and which can be easily modifiable, resulting in materials with different properties, adaptable to a wide range of applications.

To our knowledge, there are no uses of these materials for ocular permeation studies of drugs while engineered hydrogels to replicate some properties of the native corneal stroma are applied in different fields, such as tissue engineering, drug delivery systems or corneal transplantation.

The collected data from permeation studies of the four model molecules show that PVA/cellulose and chitosan hydrogels can be ideal materials for a corneal stroma model useful for evaluating drug permeation, although chitosan exhibited lower tensile strength, perhaps because of its higher water content. On the other hand, PVA/MCC-7 hydrogels displayed very similar properties to the corneal stroma such as elasticity, tensile strength, water content at physiological conditions and wettability.

However, as these methods and materials are still in the research and development stage, further studies to progress towards their possible application as functional stroma-like materials in ocular permeation studies deserve to be investigated. Our research is now aimed at the possibility of stratification of corneal epithelial cells above them in order to have a construct that simulates the corneal barrier in a more complete way.

## Figures and Tables

**Table 1:** Hydrogel compositions and methods of preparation.

Hydrogel	Component Ratios or Concentration		Method of Preparation
PVA/MCC-7	PVA:MCC	90:10	Freezing/Thawing 7 cycles
		80:20	
		70:30	
PVA/MCC-14	PVA:MCC	90:10	Freezing/Thawing 14 cycles
		80:20	
		70:30	
PVA/GEL-7	PVA:GEL	90:10	Freezing/Thawing 7 cycles
		80:20	
		70:30	
PVA/GEL-14	PVA:GEL	90:10	Freezing/Thawing 14 cycles
		80:20	
		70:30	
CHIT	Chitosan	4% *w/w*	Dissolution and Alkalization
GEL	Gelatin:glucose:water	2:1:2	UV irradiation

**Table 2:** Conditions for HPLC analysis of beta blocker drugs under study.

Molecule	Mobile Phase	Flow (mL/min)	Wavelength (nm)
Atenolol	10:90 Acetonitrile:Buffer (pH 3.8)	1.0	274
Timolol	50:50 Methanol:Buffer (pH 3.0)	1.0	294
Betaxolol	50:50 Methanol:Buffer (pH 3.0)	1.0	221
Penbutolol	70:30 Methanol:Buffer (pH 3.8)	1.0	270

Buffer = 20 mM Na_2_HPO_4_ acidified at required pH with H_3_PO_4_.

**Table 3:** Mass loss and water content percentage (mean ± SE; *n* = 3) of the hydrogels under study.

Hydrogel		Mass Loss Percentage	Water Content Percentage
PVA/MCC-7	90:10	2.34 ± 1.07	83.96 ± 0.43
	80:20	3.31 ± 0.59	83.48 ± 0.40
	70:30	3.71 ± 0.07	82.65 ± 0.61
PVA/MCC-14	90:10	−0.59 ± 1.53	84.34 ± 0.72
	80:20	2.19 ± 0.57	82.49 ± 3.46
	70:30	1.19 ± 0.69	84.90 ± 1.77
PVA/GEL-7	90:10	14.35 ± 2.55	-
	80:20	25.77 ± 0.94	-
	70:30	35.48 ± 1.16	-
PVA/GEL-14	90:10	13.34 ± 2.50	-
	80:20	25.30 ± 1.15	-
	70:30	33.60 ± 1.52	-
CHIT	-	2.10 ± 1.22	89.74 ± 0.69 *

* Statistically different from all others.

**Table 4:** Contact angle measurements (mean ± SE; *n* = 10).

Hydrogel		Contact Angle (°)
PVA/MCC-7	90:10	42.42 ± 0.70 (1)
	80:20	35.89 ± 0.59 (2)
	70:30	33.13 ± 0.60 (3)
PVA/MCC-14	90:10	41.72 ± 0.64 (4)
	80:20	37.19 ± 0.54 (5)
	70:30	34.50 ± 0.40 (6)
CHIT	-	35.66 ± 0.36

Statistically different: 1, 2, and 3 from each other; 4, 5, and 6 from each other.

**Table 5:** Enthalpy of fusion (ΔH) and temperature peak obtained by differential scanning calorimetry (mean ± SE; *n* = 3).

Hydrogel		ΔH (J/g)	Temperature (°C)
PVA/MCC-7	90:10	81.14 ± 0.60	229.1 ± 0.5
	80:20	70.46 ± 3.37	228.0 ± 1.0
	70:30	63.43 ± 0.50	228.6 ± 1.7
PVA/MCC-14	90:10	70.27 ± 0.28	230.1 ± 0.8
	80:20	69.57 ± 0.04	230.2 ± 0.4
	70:30	63.97 ± 4.38	230.4 ± 0.4

**Table 6:** Tensile strength and Young’s modulus (mean ± SE; *n* = 10).

Hydrogel		Tensile Strength (kPa)	Young’s Modulus (kPa)
PVA/MCC-7	90:10	249.10 ± 8.56	84.21 ± 3.23
	80:20	386.76 ± 21.61	146.41 ± 9.92
	70:30	638.21 ± 34.12	182.70 ± 9.18
PVA/MCC-14	90:10	689.69 ± 55.77	356.13 ± 34.86
	80:20	428.92 ± 35.41	273.70 ± 20.79
	70:30	390.03 ± 55.97	142.34 ± 18.28
CHIT	-	163.89 ± 32.20 *	110.51 ± 19.33

* Statistically different from all others.

**Table 7:** Permeability characteristics of hydrogels (mean ± SE; *n* = 5 for permeability coefficient, *n* = 3 for specific water content).

Hydrogel		Permeability Coefficient × 10^14^ (cm^2^)	Specific Water Content	Pore Size (nm)
PVA/MCC-7	90:10	2.72 ± 0.32	0.93 ± 0.06	4.82
	80:20	5.90 ± 0.56	0.95 ± 0.10	7.05
	70:30	6.61 ± 0.60	0.98 ± 0.03	7.34
PVA/MCC-14	90:10	64.61 ± 15.02	0.98 ± 0.06	22.96
	80:20	53.47 ± 19.55	0.94 ± 0.24	21.29
	70:30	31.00 ± 4.11	0.91 ± 0.09	16.48
CHIT	-	4.20 ± 1.02	1.36 ± 0.13 *	4.96

* Statistically different from all others.

**Table 8:** Physicochemical properties of the beta-blocking drugs under study.

Molecules	Molecular Weight (Da)	Partition Coefficient ^a^ (Log P)
Atenolol	266.3	0.286
Betaxolol	307.4	0.454
Penbutolol	291.4	4.121
Timolol	316.4	1.600

^a^ 1-octanol/water partition as mean value from literatures [32,52,53,54,55].

**Table 9:** Permeation parameters of beta blockers through the different hydrogels (mean ± SE; *n* = 3).

Hydrogel	P_app_ × 10^2^ (cm/h)				Slope	R^2^
	Atenolol	Betaxolol	Penbutolol	Timolol		
PVA/MCC-7 90:10	4.66 ± 0.261	4.261 ± 1.396	8.815 ± 1.246	5.344 ± 0.367	0.078 ± 0.009	0.97
PVA/MCC-7 80:20	3.654 ± 0.733	4.177 ± 0.583	8.364 ± 0.836	4.691 ± 0.569	0.089 ± 0.009	0.98
PVA/MCC-7 70:30	4.436 ± 2.615	1.523 ± 0.551	7.197 ± 0.960	5.708 ± 1.305	0.113 ± 0.088	0.45
CHIT	3.251 ± 0.782	3.960 ± 0.352	0.802 ± 0.183	2.357 ± 0.277	−0.172 ± 0.020	0.97

## Data Availability

Not applicable.
